# Morphology of the Larval Antennae and Mouthparts in *Conogethes punctiferalis* (Guenée) (Lepidoptera: Crambidae) with Special Reference to Sensilla

**DOI:** 10.3390/insects17030345

**Published:** 2026-03-21

**Authors:** Chao Yue, Shang Shi, Yaqian Shi, Peiyu Chen, Ting Lei, Na Ma

**Affiliations:** 1 Henan Key Laboratory of Insect Biology in Funiu Mountain, College of Water Resources and Modern Agriculture, Nanyang Normal University, Nanyang 473061, China; ivy_office2020@163.com (S.S.); leiting@nynu.edu.cn (T.L.); 2 Shenzhen Academy of Inspection and Quarantine, Shenzhen Custom, Shenzhen 518100, China; shiyaqian0822@163.com; 3 Nanyang Academy of Sciences, Nanyang 473083, China; chenpeiyu1984@163.com

**Keywords:** ultrastructure, larvae, sensillum, antenna, *Conogethes punctiferalis*, mouthparts

## Abstract

The yellow peach moth, *Conogethes punctiferalis*, is a destructive polyphagous pest and poses a severe threat to the fruit industry and field crops worldwide with its continuously increasing population and expanding host range in recent years. Despite the severe damage caused by *C. punctiferalis* larvae, their antennae and mouthparts, equipped with abundant sensilla responsible for feeding behavior, have not been investigated in detail. In our study, the antennae, mouthparts, and associated sensilla of first-instar and mature larvae of *C. punctiferalis* were examined with light and scanning electron microscopy. Our results revealed no obvious morphological differences between the two instars in the basic composition of the antennae and mouthparts, or in the types, distribution, and numbers of sensilla. The antenna is three-segmented, with no sensilla on the scape, three sensilla basiconica and two sensilla chaetica on the pedicel, and three sensilla basiconica and one sensillum styloconicum on the flagellum. The mouthparts of *C. punctiferalis* are typically mandibulate and consist of a labrum-epipharynx, paired mandibles, a pair of maxillae, a labium, and a hypopharynx. Six types of sensilla were primarily concentrated on the labrum-epipharynx, maxilla, and labial palp, including sensilla chaetica, sensilla basiconica, sensilla styloconica, sensilla digitiformia, sensilla epipharyngea, and sensilla placodea. We conducted a systematic analysis of the characteristics of sensilla and discussed their variation in the context of Lepidoptera phylogeny. The potential functions of the sensilla have also been inferred. The study could advance our understanding of the behavioral ecology of *C. punctiferalis* and provide potentially useful information on the development of pest control technologies.

## Simple Summary

*Conogethes punctiferalis* is a well-known, highly polyphagous pest that poses a serious threat to agriculture and forestry across numerous countries. As important sensory and feeding organs, the larval antennae and mouthparts of *C. punctiferalis* have received little research attention to date. In this study, the ultrastructure of the antennae, mouthparts, and associated sensilla of *C. punctiferalis* larvae was investigated using light and scanning electron microscopy. We conducted a systematic analysis of the characteristics of sensilla and discussed their variation in the context of Lepidoptera phylogeny. The potential functions of these sensilla have also been inferred.

## 1. Introduction

The antennae and mouthparts equipped with abundant sensilla are intricate structures of the insect head and play significant roles in ecological behaviors, such as host plant detection, feeding, and mating [1]. They have diversified tremendously in both morphology and function across insect groups through evolution [2]. Sensilla are cuticular structures of varying size and shape, specialized for the perception of various stimuli (e.g., taste, smell, temperature, and humidity) [3]. In recent years, the sensilla, together with the structures of antennae and mouthparts, have gained considerable attention, serving as a foundation for both pest management and taxonomic studies [4,5,6,7,8].

The yellow peach moth, *Conogethes punctiferalis* (Guenée, 1854) (Lepidoptera: Crambidae), is a well-known highly polyphagous pest [9]. As a pest of global significance in agricultural plants, they are not only distributed across the Australasian region from India to New Guinea, the Solomon Islands, and Australia, but also found in Hawaii and Great Britain [10]. Previous studies have documented that larvae can attack over 120 species, including crops, fruits, vegetables, and spices, causing serious losses in yield and quality [10,11,12]. In China, *C. punctiferalis* has replaced the Asian corn borer, *Ostrinia furnacalis* (Guenée), as the most destructive pest in maize due to the changes in climate and cropping systems [13,14]. It exhibits a shift in host choice between generations and, as a boring pest spending the whole life cycle inside hosts, is less susceptible to pesticides [10,15,16]. With its continuously increasing population and expanding host range in recent years, *C. punctiferalis* poses a severe threat to the fruit industry and field crops in temperate to tropical regions worldwide [9,10].

Like most lepidopteran pests, *C. punctiferalis* causes damage primarily through its larval feeding and development within both vegetative and reproductive plant tissues [10]. The antennae and mouthparts of the larvae, equipped with various sensilla, are critical for chemical detection, regulating host plant recognition and feeding preferences [17,18,19]. Therefore, studying the morphology of these appendages, as well as the types, distribution, and numbers of their associated sensilla, is essential for understanding their feeding behaviors and the underlying mechanisms [7]. The larval sensilla of the Lepidoptera have been extensively studied in many species such as *Homoeosoma nebulella* and *Ephestia kuehniella* of Pyralidae [20,21], *Olethreutes Cespitana* and *Grapholita molesta* of Tortricidae [22,23], *Agrotis ypsilon* of Noctuidae [24], *Tuta absoluta* of Gelechiidae [17], *Plutella xylostella* of Plutellidae [25], *Carposina sasakii* of Carposinidae [26], *Dendrolimus kikuchii* of Lasiocampidae [27], and *Chilo infuscatellus* of Crambidae [8]. No related studies have been conducted on the larvae of *C. punctiferalis*.

The aim of this study is to characterize the morphology of the larval antennae and mouthparts of both first-instar and mature larvae of *C. punctiferalis*, as well as the associated sensilla, including their types, distribution, and numbers, with light and scanning electron microscopy. The results could offer a basis for behavioral ecology and pest management practices.

## 2. Materials and Methods

The larvae of *C. punctiferalis* were collected in chestnut orchards (*Castanea mollissima* Blume) in Tongbai County, Henan Province, in September 2022. The larvae, together with the infested fruits, were brought back to the laboratory and placed in transparent plastic boxes covered with a perforated lid. The larvae were reared in a growth chamber at 25 ± 1 °C, 75 ± 5% RH, and 16:8 LD till adult emergence. Freshly emerged adults were released into the insect rearing cage (35 cm × 45 cm × 20 cm), which is provided with degreasing cotton balls saturated with 10% sugar solution in a glass dish (10 cm in diameter) for mating and oviposition. The cage was covered with a piece of gauze on the top as an oviposition substrate. Eggs were collected daily and incubated in growth chambers. After hatching, the first instar larvae were transferred to the transparent plastic boxes and reared on fresh chestnut fruit for multiple successive generations. The first-instar and mature larvae were collected for light and scanning electron microscopy investigation.

For light microscopy, more than 10 larvae from each of the two developmental stages were randomly selected and killed by immersion in 60 °C water to straighten and expand the body, and preserved in 75% ethanol at 4 °C until use. Photographs were taken with a QImaging Retiga 2000R Fast 1394 Digital CCD camera (QImaging, Surrey, BC, Canada) attached to a Nikon SMZ1500 Stereoscopic Zoom Microscope (Nikon, Tokyo, Japan). Voucher specimens were deposited in the zoological specimen museum of Nanyang Normal University (NYNU). For scanning electron microscopy, the larvae at both stages were anesthetized in ethyl ether and decapitated quickly. The heads were immediately fixed in Carnoy’s fixative solution (95% ethanol: glacial acetic acid = 3:1 *v*/*v*) for 12 h at 4 °C to preserve fine morphological structures. After fixation, the specimens were preserved in 75% ethanol for long-term storage at 4 °C until further processing. For the mature larvae, the antennae and each mouthpart component were dissected under a Nikon SMZ1500 Stereoscopic Zoom Microscope and cleaned in an ultrasonic bath for 30 s in 75% ethanol. Ten first-instar specimens and 20 mature larvae were used for scanning electron microscopy. Subsequently, they were dehydrated through a graded ethanol series (80%, 90%, 95%, and 100% twice). The ethanol was then replaced with graded mixtures of ethanol and tertiary butanol (3:1, 1:1, and 1:3, *v*/*v*) for 15 min each, followed by two treatments with pure tertiary butanol for 30 min each. Then the samples were freeze-dried for 3 h. After sputter-coating with gold, the samples were examined under a Hitachi S-3400N scanning electron microscope (Hitachi, Tokyo, Japan) at 15 kV.

Sensilla were named according to the nomenclature used in previous studies on lepidopterous larvae [17,26,28]. The length and basal diameter of the sensilla of mature larvae were measured using ImageJ version 15.4g. The data are presented as mean ± standard deviation (sd). Individual sensilla that were damaged, obscured, or abnormally positioned were excluded from measurement. The sample size (*n*) represents the maximum number of individual sensilla available for morphological measurement in the study. Raw data for individual sensilla measurements are presented in Supplementary Table S1 (Excel format).

## 3. Results

### 3.1. General Morphology of the Head

**Figure 1:**
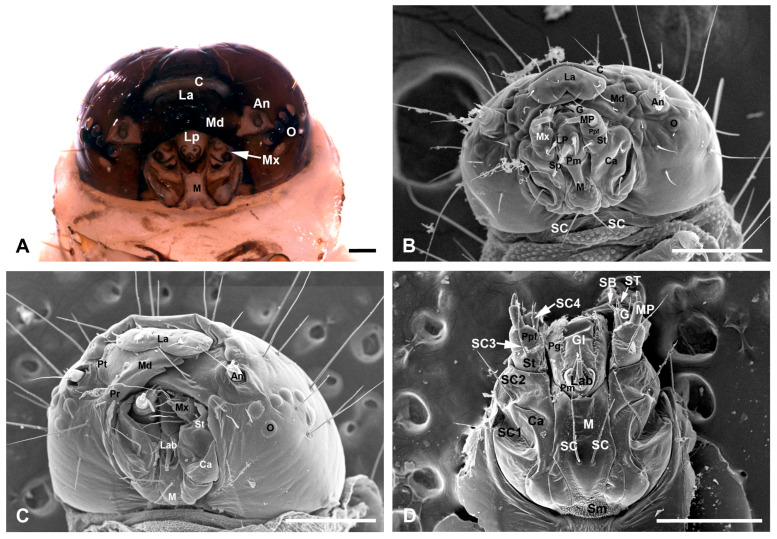
Light and scanning electron micrographs of the larval heads of *C. punctiferalis*. (**A**) Head of the mature larva, ventrofrontal view. (**B**) Head of the first-instar larva, ventrofrontal view. (**C**) Head of the mature larva, ventrofrontal view. (**D**) Maxilla and Labium of the mature larva, posterior view. An, antenna; C, clypeus; Ca, cardo; G, galea; Gl, glossae; La, labrum; Lab, labium; LP, labial palp; M, mentum; Md, mandible; Mx, maxilla; MP, maxillary palp; O, ocellus; Pg, paraglossa; Pm, prementum; Pr, precoila, Pt, postcoila, Ppf, palpiger; Sm, submentum; Sp, spinneret; St, stipes; SB, sensilla basiconica; SC, sensilla chaetica; ST, sensilla styloconica. Scale bars: (**A**) = 200 μm; (**B**) = 100 μm; (**C**,**D**) = 500 μm.

**Figure 2:**
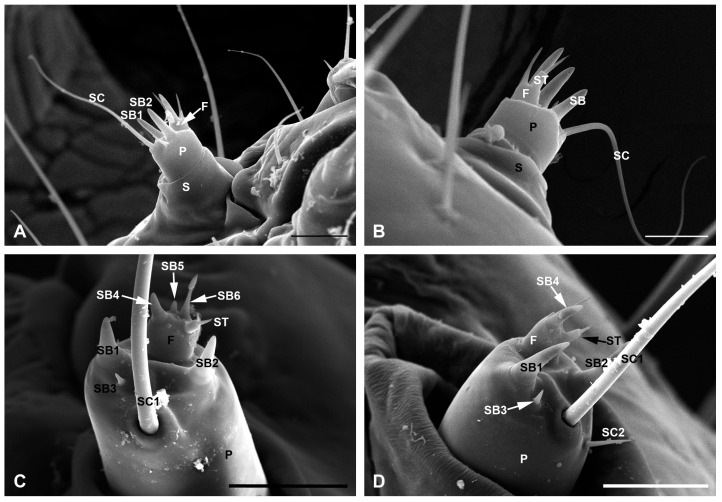
Antennae of *C. punctiferalis* larvae. (**A**,**B**) Ventral and dorsal views of the right antennae of the first-instar larva. (**C**,**D**) Lateral and dorsal views of the left antennae of the mature larvae. F, flagellum; P, pedicel; S, scape; SB, sensilla basiconica; SC, sensilla chaetica; ST, sensilla styloconica. Scale bars: (**A**) = 20 μm; (**B**) = 15 μm; (**C**,**D**) = 50 μm.

The head of the *C. punctiferalis* is highly sclerotized and hypognathous, possessing the main sensory organs on the frontal portion, such as the antennae, mouthparts, and six pairs of stemmata (Figure 1). In addition to the scattered seta on the head capsule, the sensilla of the larval head are mainly distributed on the antennae and mouthparts (Figure 1C). The paired antennae are short and arise from a prominent, membranous, approximately triangular region between the arcs formed by the six stemmata and mouthparts (Figure 1A). Each antenna consists of three segments: a scape, a pedicel, and a flagellum (Figure 2A,B). The mouthparts are mandibulate and consist of a labrum-epipharynx, a pair of mandibles, a pair of maxillae, a labium, and a hypopharynx (Figure 1A). The maxillae and labium form a maxilla-labial complex (Figure 1B–D). No obvious morphological differences were observed between the first-instar and mature larvae, either in the basic composition of the antennae and mouthparts, or in the features of associated sensilla. The only differences were in overall size and degree of development (Figure 1B,C and Figure 2). Due to the small size and difficulty in dissecting the head of first-instar larvae, we only measured the major sensilla on the head of mature larvae in this study. The mean length and basal diameter of the sensilla on the mature larval antenna and mouthparts are listed in Table 1 and Table 2, respectively.

### 3.2. The Antennae

Among the three antennal segments, the scape is the widest and devoid of any sensilla (Figure 2A,B). The cylindrical pedicel is thinner than the scape. Its length-to-width ratio is much smaller in first-instar larvae than in mature larvae (Figure 1B,C). Two sensillum types are present on the pedicel: three sensilla basiconica (SB1–SB3) at the median region and two sensilla chaetica (SC1 and SC2) on the outer side margin (Figure 2C,D). Among the three sensilla basiconica, SB1 (29.71 ± 7.20 μm long and 12.98 ± 0.63 μm wide) is similar to SB2 (32.37 ± 5.82 μm long and 11.71 ± 1.73 μm wide), but slightly longer and stouter. They are situated on the outer margin of the pedicel top near the flagellum. SB3 (7.62 ± 2.03 μm long and 3.57 ± 0.51 μm wide) is markedly shortest and thinnest, located between SB1 and SB2 and closer to SB1 (Figure 2C,D). SC1 and SC2 are remarkably different in both length and width: SC1 (319.39 ± 93.31 μm long and 8.06 ± 0.51 μm wide) is much longer and thicker than SC2 (24.71 ± 4.31 μm long and 3.27 ± 0.35 μm wide). SC1 and SC2 are both located on the most ventral margin of the pedicel apex. The flagellum is short and located on the dorsal margin of the pedicel. Three sensilla basiconica (SB4–SB6) and one sensillum styloconicum (ST) are arranged around the apical margin (Figure 2C,D). SB4 (24.45 ± 4.16 μm long and 8.39 ± 0.58 μm wide) is longer and thicker than SB6 (19.92 ± 1.83 μm long and 4.28 ± 0.89 μm wide). SB5 is located between SB4 and SB6, with the shortest length of 4.05 ± 1.44 μm and a width of 2.22 ± 0.86 μm. Situated in the ventral margin of the flagellum, the sensillum styloconicum is an elongated sensory cone (7.83 ± 1.27 μm long and 1.66 ± 0.41 μm wide) inserted at the tip of a columnar projection (7.05 ± 1.73 μm long and 4.62 ± 0.22 μm wide) (Figure 2C,D).

### 3.3. The Mouthparts

#### 3.3.1. The Labrum-Epipharynx

**Figure 3:**
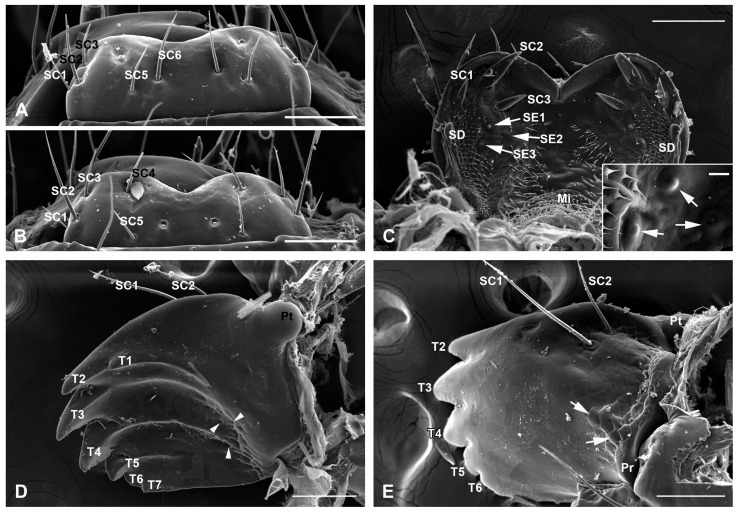
Labrum-epipharynx and mandibles of the mature larvae of *C. punctiferalis*. (**A**,**B**) Dorsal view of the labrum. (**C**) The epipharynx, with an inset (scale bar = 10 μm) showing magnified sensilla epipharynges from a different specimen. (**D**,**E**) Internal and external view of the left mandible. Arrowheads and arrows show the pores on the internal surface and scale-like structures on the external surface, respectively. Mi, microtrichia; Pr, precoila, Pt, postcoila, SC, sensilla chaetica; SD, sensilla digitiformia; SE, sensilla epipharynges; T, tooth. Scale bars = 150 μm.

The labrum is broad and highly chitinized, with the basal part connected to the membranous clypeus (Figure 1A,B). The apical margin is blunt and equipped with a broad V-shaped notch in the middle region (Figure 3A,B). Only one type of sensillum, sensilla chaetica, was found on the labrum. The six pairs of sensilla chaetica (SC1–SC6) on the labrum surface, with varying lengths, are distributed symmetrically along the median longitudinal line. SC1–SC3 are arranged in sequence along the lateral margin, while SC5 and SC6 are located in the middle region. SC4 is situated between these two groups, adjacent to the apical margin (Figure 3A,B). From the measurement statistics, SC2 (193.74 ± 23.67 μm long and 9.51 ± 0.81 μm wide) is the longest and thickest, while SC1 (80.47 ± 9.52 μm long and 5.54 ± 0.88 μm wide) is the shortest and thinnest (Table 2).

The epipharynx is situated on the inner surface of the labrum and symmetrically furnished with three types of sensilla: three pairs of sensilla chaetica (SC1–SC3), three pairs of sensilla epipharyngea (SE1–SE3), and two sensilla digitiformia (SD) (Figure 3C). SC1–SC3 are thick and flattened, forming a triangular arrangement at the apicolateral corners created by the emargination. Among them, SC1 is the shortest and thinnest, located near the lateral margin, with 54.42 ± 13.29 μm long and 16.71 ± 2.86 μm wide. SC3 is much closer to the middle area, with an intermediate length of 70.44 ± 10.27 μm and a width of 17.99 ± 1.61 μm. Compared to the former two, SC2 is the longest and thickest, with a length of 89.16 ± 12.91 μm and a width of 22.99 ± 3.27 μm. Due to its length and proximal position to the apical margin, it is readily visible in the ventrofrontal view (Figure 1B,C). Three paired SE (SE1, 13.70 ± 2.76 μm wide; SE2, 12.57 ± 1.41 μm wide; SE3, 20.94 ± 3.05 μm wide) are distributed on the glabrous surface of the epipharynx. The SD (83.37 ± 8.48 μm long and 22.36 ± 3.36 μm wide) extends along each lateral margin of the epipharynx, exhibiting an expansion in its apical third (Figure 3C). Numerous microtrichia formed by the cuticular processes cover the basal region and extend forward, forming two lateral bands and two median bands. The space between bands is glabrous (Figure 3C).

#### 3.3.2. The Mandibles

The paired mandibles are highly sclerotized and roughly symmetrical, articulated with the pleurostomal region via their precoila and postcoila. Under natural conditions, the paired mandibles are overlapped for more than half their length, with most of their area in frontal view not covered by the labrum (Figure 1B,C). The apical cutting edge of the mandible bears seven incisor cusps, among which T1 and T7 are quite short, T2–T5 are distinctly longer, stronger, and sharply pointed, and T6 is short and broad, with a blunt tip (Figure 3D,E). The molar region is completely reduced, with the grinding surface absent, but some pores are present on the basal anterior part of the internal surface (Figure 3D). The external surface of each mandible is glabrous, with scale-like structures confined to the basal region and two long sensilla chaetica (SC1 and SC2) located at the basal-posterior region (Figure 3E). SC2 (166.67 ± 12.53 μm long and 9.37 ± 0.42 μm wide) is much closer to the margin and shorter than SC1 (279.34 ± 21.16 μm long and 10.94 ± 0.28 μm wide).

#### 3.3.3. The Maxillae

**Figure 4:**
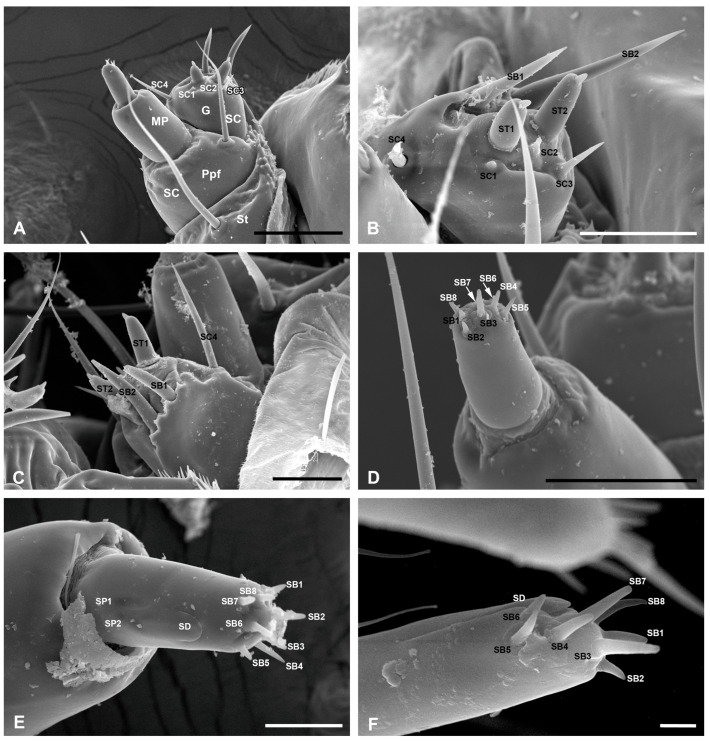
Sensilla on the maxilla of *C. punctiferalis* larvae. (**A**) The right maxilla of the mature larva, ventral view. (**B**) The galea of the mature larva, ventral view. (**C**) The galea of the mature larva, dorsal view. (**D**) The maxillary palp of the mature larva, showing the sensilla on the apex. (**E**) Inner side of the maxillary palp of the mature larva. (**F**) The distal segment of the maxillary palp of the first instar. G, galea; MP, maxillary palp; Ppf, palpiger; St, stipes; SB, sensilla basiconica; SC, sensilla chaetica; SD, sensilla digitiformia; SP, sensilla placodea; ST, sensilla styloconica. Scale bars: (**A**) = 100 μm; (**B**–**D**) = 50 μm; (**E**) = 20 μm; (**F**) = 2 μm.

The paired maxillae are located beneath the mandibles and roughly symmetrical, each consisting of a basal cardo, a middle stipes, a galea, and a maxillary palp (Figure 1B,D). The cardo is broad and predominantly membranous, with only the median region being sclerotized (Figure 1A). It is elongate, occupying nearly two-thirds of the length of the maxillae. Two long sensilla chaetica (SC1, 342.08 ± 43.75 μm long and 14.07 ± 1.84 μm wide; SC2, 235.54 ± 31.35 μm long and 10.41 ± 1.43 μm wide) are located above and below this sclerotized region, respectively. The stipes are short and bear a sensillum chaeticum (SC3) on their median part, with a length of 268.22 ± 44.35 μm and a width of 10.38 ± 1.19 μm (Figure 1C,D). The inner lateral surface of the stipes is furnished with numerous granular cuticular processes and connected apically to the galea (Figure 4A). The palpifer is membranous and fused with the distal margin of the stipes, bearing a sensillum chaeticum (SC4, 127.88 ± 10.28 μm long and 7.89 ± 1.13 μm wide) on its inner side (Figure 1A,D), which is much shorter and thinner than the sensilla chaetica described above.

The galea is equipped with three types of sensilla, including two sensilla basiconica (SB1 and SB2), two sensilla styloconica (ST1 and ST2), and four sensilla chaetica (SC1–SC4) (Figure 4A–C). SB1 and SB2 with sharp tips are located on the dentate ventral margin of the galea apex (Figure 4C). The inner one, SB2 (65.61 ± 7.33 μm long and 10.21 ± 1.50 μm wide), is much longer and thicker than SB1 (51.81 ± 7.60 μm long and 8.57 ± 1.19 μm wide). ST1 and ST2, each with a conical tip and a strongly elevated socket, are similar in size and distributed in the central region of the apex (Figure 4B, Table 2). SC1–SC3 are situated on the galea apex, while SC4 is located on the inner lateral side of the galea (Figure 4A,B). They vary remarkably in length: SC4 is significantly longer than the other three, with its length of 70.25 ± 13.78 μm and width of 6.14 ± 1.18 μm. In contrast, SC1 is the shortest, as it is 4.84 ± 1.19 μm long and 5.19 ± 1.29 μm wide. SC2 (12.21 ± 3.90 μm long and 7.04 ± 2.11 μm wide) is located close to the paired sensilla styloconica. SC3 (25.68 ± 4.08 μm long and 5.54 ± 1.27 μm wide) bears a sharp tip and is approximately twice the length of SC2 (Figure 4A,B, Table 2).

The maxillary palp is two-segmented and inserted on the palpifer. The basal segment is thick and cylindrical, lacking sensilla. The distal segment is much thinner and shorter, furnished with a cluster of short sensilla basiconica (SB1–SB8) at its apex. SB1–SB8 rise from sockets and vary in size, with SB1 (5.31 ± 2.28 μm long and 2.03 ± 0.64 μm wide) being the shortest and thinnest, whereas SB3 (8.68 ± 2.20 μm long and 2.48 ± 0.31 μm wide) is the longest and thickest (Figure 4D, Table 2). A sensillum digitiformium (SD), located on the inner lateral surface of the maxillary palp, exhibits considerable morphological differences between the first-instar and mature larvae. In the first-instar larvae, it projects to the apex as a finger-like extension embedded in a cuticular groove, whereas in mature larvae it is flattened and ends subapically in a fingernail-like shape (Figure 4E,F). Two inconspicuous sensilla placodea (SP1 and SP2) are situated within a cuticular depression beneath the SD. Each sensillum features a distinct oval convexity with a smooth surface. The depression is bordered by a slightly elevated cuticle rim (Figure 4E).

#### 3.3.4. The Labium

**Figure 5:**
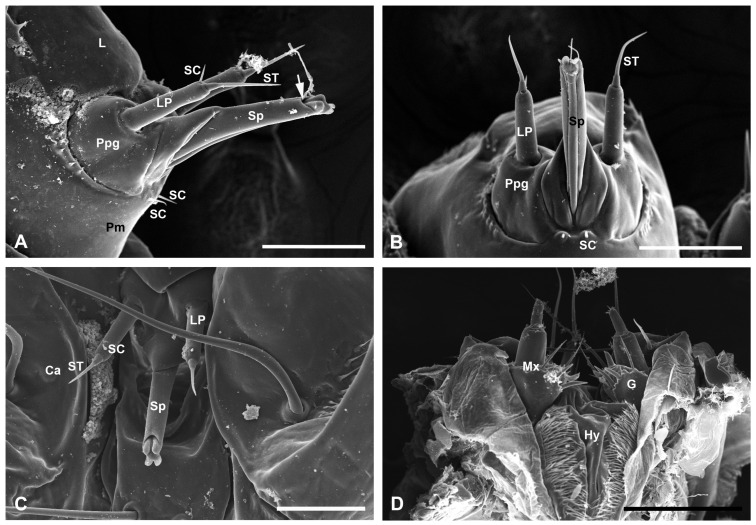
The labium and hypopharynx of *C. punctiferalis* mature larvae. (**A**–**C**) Lateral, posterior, and frontal views of the labium, with the arrow indicating the apical pore. (**D**) Hypopharynx. Ca, cardo; G, galea; Hy, hypopharynx; L, lingula; LP, labial palp; Mx, maxilla; Ppg, palpiger; Pm, prementum; SC, sensilla chaetica; Sp, spinneret; ST, sensilla styloconica. Scale bars = 100 μm.

The labium consists of the basal postmentum and the distal prementum. The postmentum, situated between the paired cardines of the maxillae, is subdivided into the basal submentum and the distal mentum (Figure 1B,D). In the mature larvae, the submentum is densely covered with microtrichia and occupies only the basal portion of the postmentum, while the mentum is elongated and predominantly membranous, with only the lateral and apical margins being sclerotized (Figure 1A,D). It is worth noting that the mentum exhibits marked morphological differences between the first-instar and mature larval stages. In the first instar larvae, the mentum is distinctly convex and glabrous centrally, bearing only a pair of sensilla chaetica on the uneven basal region (Figure 1B). In contrast, in the mature larvae, the mentum is only slightly convex in the basal half. Its surface is furnished with short microtrichia on both lateral regions and two strong sensilla chaetica (249.91 ± 38.76 μm in length and 11.40 ± 0.77 μm in width) on the median convex region (Figure 1D). The prementum, separated from the postmentum by a deep fold, bears a pair of labial palps, the mesal glossae, and the lateral paraglossae (Figure 1B,D). Two extremely minute sensilla chaetica (SC, 25.18 ± 4.91 μm long and 3.55 ± 0.23 μm wide) are located on the apical median region of the prementum, each only about half the length of those on the mentum (Figure 5A,B, Table 2).

The paired labial palps and the median spinneret occupy the specialized distal region of the labium. The labial palps are unsegmented and situated on the chitinized palpigers. Each palp bears a sensillum styloconicum (ST) on the apex and a small, sharp sensillum chaeticum (SC, 18.58 ± 2.47 μm long and 3.32 ± 0.49 μm wide) on the dorsolateral subapex (Figure 5A–C). The ST possesses a short basal columnar socket (11.14 ± 2.66 μm long and 9.06 ± 0.91 μm wide) and an elongated slender rod (54.02 ± 8.07 μm long and 5.21 ± 0.79 μm wide), with the rod being approximately four times longer than the basal socket. The spinneret protrudes from the mesal surface of the paired palpigers. The base of the spinneret is chitinized and appears triangular in the lateral view and roughly rhombic in the ventral view (Figure 1A and Figure 5A,B). In the external view, the spinneret appears as a tubular structure comprising a dorsal sheath and a ventral columnar structure, with a four-lobed projection extending from its apex (Figure 5B,C). The spinneret secreted the silk from the dorsal-apical pore (Figure 5A,B).

The mesal glossae are glabrous and entirely fused with each other, forming a single structure that is basally united with the paired lateral paraglossae. The paraglossae are furnished with dense microtrichia along the mesolateral margins. They constitute the membranous lingula, which forms the floor of the preoral cavity along with the prementum (Figure 1D).

#### 3.3.5. The Hypopharynx

The hypopharynx is a pouch-like structure located in the preoral cavity. In the dorsal side, the median region is swollen and hairless, while the lateral regions are folded and furnished with dense and inwardly directed microtricha (Figure 5D).

## 4. Discussion

In recent years, *C. punctiferalis* has shown a trend of aggravating infestation and expanding host range, posing a serious threat to global agriculture and forestry [9,10,29,30]. As important sensory and feeding organs, antennae and mouthparts of *C. punctiferalis* have only been documented in adults [31]. Because the larval stage represents the most destructive period in the life cycle, the present study describes the morphology of the antennae and mouthparts of larval *C. punctiferalis*, with a focus on their associated sensilla. Based on a preliminary comparison of the overall morphology of the antennae and mouthparts between the first-instar and mature larvae, we found that only the pedicel (length-to-width ratio), the sensillum digitiformium on the maxillary palp, and the mentum exhibited slight differences. Their basic composition, as well as the types, distribution, and numbers of sensilla, showed no obvious morphological differences between the two instars, apart from differences in overall size and developmental degree. Our findings are consistent with those in *Spodoptera frugiperda* (Noctuidae) [32]. It is speculated that the larger sensilla in older larvae may improve perception of a complex environment and enable behavioral responses beneficial to their survival [7,32]. For *C. punctiferalis*, since its entire larval stage is spent idle and feeding within the host fruit, the sensilla increase only in size as the larva grows, with no change in either type or number.

Positioned prominently at the front, the antennae serve as the primary interface with the environment [2]. As the most crucial organ for odor detection, the antenna houses various sensilla and plays key roles in foraging [32]. Based on the previous studies, we found that the types, distribution, and numbers of sensilla on the antennae are largely consistent across different lepidopteran larvae, although some description discrepancies that occurred in certain species may arise from variations in equipment or inconsistencies in terminology [17,20,21,25,26,33]. The antennal sensilla of *C. punctiferalis* studied here follow the general pattern of lepidopteran larvae, comprising six sensilla basiconica, two sensilla chaetica, and one sensillum styloconicum on each antenna. In Cossidae, however, the antenna bears eight sensilla basiconica in total, a notable increase in number compared to the typical pattern [34]. An increase in the number of sensilla within receptor groups has been regarded as a progressive evolutionary trend, which may enhance the ability to perceive environmental information [35]. However, given the phylogenetic position of this family within Lepidoptera, this increased number cannot be interpreted as a synapomorphy. This is because even basal lineages within the Apoditrysia clade, such as Tortricidae and Plutellidae, still possess the typical six sensilla basiconica [36]. Among sensilla types on lepidopteran larval antennae, sensilla basiconica are usually the most abundant and commonly function as olfactory organs for detecting plant volatiles [37]. The eight sensilla basiconica may represent an autapomorphy of Cossidae, possibly associated with their trunk host-transfer behavior [34]. Sensilla styloconica, which are considered a type of cold-sensitive receptor [38,39], display substantial variation in number among the antennal sensilla of lepidopteran larvae. In the early shoot borer *Chilo infuscatellus*, a destructive stem-boring pest of sugarcane in southern China, the sensilla styloconica are absent [8]. This morphological absence could be an adaptive trait, as this species inhabits humid subtropical climate zones with consistently mild temperatures, thus possibly rendering specialized cold-sensing apparatus superfluous [8,38,39]. Conversely, two sensilla styloconica were observed in two other lepidopteran species, *Chilo partellus* and *Maruca vitrata*, both of which are boring pests within Pyraloidea [4]. This quite differs from both other larvae within the same taxonomic group and those with similar boring habits [17,22,26]. Therefore, we suggest that the observed variations in the number of this sensillum type cannot be explained by phylogeny or feeding habits. In the study of *S. frugiperda*, sensilla campaniform and small pores were reported on the third and fifth instar larval antennae, which may be associated with their rapid dispersal ability and adaptation to diverse environments [32].

The mouthparts of Lepidoptera larvae are generally composed of a labrum-epipharynx, paired mandibles, and a maxilla–labial complex [28]. Our study revealed that the sensilla of *C. punctiferalis* mouthparts were primarily concentrated on the labrum–epipharynx, maxilla, and labial palp. The labrum-epipharynx of lepidopteran larvae is considered to be formed by the fusion of two lobes, evidenced by the notch that is usually retained on the anterior edge [28]. Generally, the labrum bears only sensilla chaetica, which are symmetrically distributed on the two lobes and vary in number from 10 to 18 among different groups [4,40]. In most cases, six pairs are arranged on the two lobes [8,38,41,42]. The epipharynx is usually furnished with three types of sensilla, including the sensilla chaetica, sensilla digitiformia, and sensilla epipharyngea. Among these sensilla, sensilla chaetica and sensilla digitiformia are remarkably consistent in number and position with those found in other lepidopteran larvae: the former occurs in three pairs located in the anterior region of the epipharynx, while the latter typically has one pair positioned on the sides of the epipharynx. The sensilla epipharyngea, presumed to have an olfactory function in sensing plant volatiles [20], show considerable variation in number across different groups, ranging from zero to three pairs, with one or three pairs being more common [8,20,25,41,42]. The mandibles function in piercing, cutting, grinding, and facilitating food ingestion, and their morphology varies across lineages with different feeding habits [3,42]. In concealed-feeding larvae (i.e., feeding inside host tissues), the mandibles are generally furnished with incisor cusps, the number of which varies among species but is typically five [42]. For example, *Carposina sasakii* of Carposinidae, *Tuta absoluta* of Gelechiidae, *Deudorix isocrates* of Lycaenidae, and *Grapholita molesta* of Tortricidae all possess five cusps [17,22,26,40]; while *C. punctiferalis* and the other species, *Chilo infuscatellus*, both belonging to Crambidae, bear seven [8]. Whether this feature can serve as a taxonomic characteristic for larvae of the family requires further investigations across a broader range of taxa.

As in most Lepidoptera larvae, the maxilla of *C. punctiferalis* is the mouthpart component with the greatest diversity of sensillum types, concentrated primarily at the apices of both the galea and maxillary palp, and follows the typical sensillar pattern observed in most lepidopteran larvae. Specifically, the galea bears three sensilla types (sensilla chaetica, sensilla basiconica, and sensilla styloconica), whereas the maxillary palp is equipped with 8 sensilla basiconica on the apex, one sensillum digitiformium on the inner lateral surface, and two inconspicuous sensilla placodea below it [4,17,20,27,33]. In some groups, the presence of one sensillum styloconicum on the maxillary palp is accompanied by the reduction in one sensillum basiconicum [21,25,26], which may reflect a functional trade-off between thermoreception and olfaction.

The mouthparts, as the feeding organs of insects, are generally equipped with various sensilla, including chemoreceptors for tasting food and mechanoreceptors for touch and positioning [2]. Lepidoptera larvae generally possess mandibulate mouthparts and exhibit mass-feeding behavior, causing severe losses to agriculture and forestry [28,42]. The diverse sensilla on their mouthparts are crucial for executing this damaging feeding process [4]. From the perspective of the overall mouthparts of *C. punctiferalis*, six types were identified: sensilla chaetica, sensilla basiconica, sensilla styloconica, sensilla digitiformia, sensilla epipharyngea, and sensilla placodea. In our study, sensilla chaetica were mainly found on the external surface of the mouthpart, such as the labrum, mandibles, components of the maxilla (cardo, stipes, galea), and the labium (mentum, prementum). These sensilla are interpreted as contact chemo-mechanoreceptors, given their exposure to contact and presumed role in sensing mechanical cues and recognizing food texture [32,43,44]. The maxilla and labial palps are considered vital organs for regulating host plant recognition and feeding preferences, with their associated sensilla styloconica and sensilla basiconica for gustatory and olfactory perception, respectively [45,46,47]. The sensilla digitiformia and sensilla placodea may be important for host-migration behavior and for selecting a suitable host, as they are considered to be chemoreceptors [48,49]. In larval behaviors such as host location, feeding, and predator avoidance, the various sensilla on the mouthparts, in concert with the antennae, play a vital role.

## Figures and Tables

**Table 1:** Length and basal diameter of different sensilla types on the antennae in the *C. punctiferalis* larval antennae.

Location	Sensillum Type	Length (μm)	*n*	Basal Diameter (μm)	*n*
Pedicel	SC1	319.39 ± 93.31	5	8.06 ± 0.51	10
	SC2	24.71 ± 4.31	8	3.27 ± 0.35	8
	SB1	29.71 ± 7.20	8	12.98 ± 0.63	8
	SB2	32.37 ± 5.82	6	11.71 ± 1.73	9
	SB3	7.62 ± 2.03	14	3.57 ± 0.51	7
Flagellum	SB4	24.45 ± 4.16	9	8.39 ± 0.58	10
	SB5	4.05 ± 1.44	12	2.22 ± 0.86	11
	SB6	19.92 ± 1.83	8	4.28 ± 0.89	7
	ST tip	7.83 ± 1.27	15	1.66 ± 0.41	13
	ST base	7.05 ± 1.73	10	4.62 ± 0.22	10

Note: Data are presented as mean ± sd. Sensilla abbreviation: SB, sensilla basiconica; SC, sensilla chaetica; ST, sensilla styloconica.

**Table 2:** Length and basal diameter of different sensilla types on the mouthparts of the *C. punctiferalis* larvae.

Location		Sensillum Type	Length (μm)	*n*	Basal Diameter (μm)	*n*
Labrum		SC1	80.47 ± 9.52	15	5.54 ± 0.88	18
		SC2	193.74 ± 23.67	15	9.51 ± 0.81	16
		SC3	107.77 ± 10.79	10	8.08 ± 1.06	17
		SC4	85.93 ± 12.07	11	8.75 ± 1.62	12
		SC5	98.09 ± 14.90	10	7.68 ± 1.15	13
		SC6	130.44 ± 17.27	8	8.45 ± 1.27	10
Epipharynx		SC1	54.42 ± 13.29	11	16.71 ± 2.86	11
		SC2	89.16 ± 12.91	11	22.99 ± 3.27	12
		SC3	70.44 ± 10.27	12	17.99 ± 1.61	12
		SE1	–	–	13.70 ± 2.76	11
		SE2	–	–	12.57 ± 1.41	8
		SE3	–	–	20.94 ± 3.05	8
		SD	83.37 ± 8.48	9	22.36 ± 3.36	15
Mandible		SC1	279.34 ± 21.16	9	10.94 ± 0.28	10
		SC2	166.67 ± 12.53	9	9.37 ± 0.42	9
Maxilla	Cardo	SC1	342.08 ± 43.75	24	14.07 ± 1.84	24
		SC2	235.54 ± 31.35	22	10.41 ± 1.43	26
	Stipe	SC3	268.22 ± 44.35	28	10.38 ± 1.19	33
	Palpifer	SC4	127.88 ± 10.28	33	7.89 ± 1.13	33
	Galea	SC1	4.84 ± 1.19	14	5.19 ± 1.29	14
		SC2	12.21 ± 3.90	15	7.04 ± 2.11	14
		SC3	25.68 ± 4.08	16	5.54 ± 1.27	16
		SC4	70.25 ± 13.78	13	6.14 ± 1.18	18
		SB1	51.81 ± 7.60	21	8.57 ± 1.19	12
		SB2	65.61 ± 7.33	20	10.21 ± 1.50	11
		ST1 base	29.18 ± 6.34	17	13.29 ± 2.83	16
		ST1 tip	6.24 ± 1.12	17	3.49 ± 0.53	16
		ST2 base	30.88 ± 7.00	15	13.66 ± 1.50	15
		ST2 tip	6.14 ± 1.09	15	3.36 ± 0.42	15
	Maxillary Palp	SB1	5.31 ± 2.28	14	2.03 ± 0.64	16
		SB2	6.89 ± 1.73	12	2.24 ± 0.33	17
		SB3	8.68 ± 2.20	17	2.48 ± 0.31	18
		SB4	7.60 ± 2.24	18	2.18 ± 0.34	19
		SB5	8.19 ± 2.69	17	2.25 ± 0.26	18
		SB6	6.12 ± 1.25	14	2.23 ± 0.50	14
		SB7	6.22 ± 1.36	10	2.35 ± 0.29	11
		SB8	7.22 ± 2.20	15	2.17 ± 0.32	13
		SD	12.36 ± 2.61	6	8.02 ± 0.26	4
		SP	10.44 ± 1.31	5	3.78 ± 0.84	5
Labium	Prementum	SC	25.18 ± 4.91	6	3.55 ± 0.23	20
	Mentum	SC	249.91 ± 38.76	23	11.40 ± 0.77	31
	Labial Palp	SC	18.58 ± 2.47	10	3.32 ± 0.49	18
		ST base	11.14 ± 2.66	24	9.06 ± 0.91	25
		ST tip	54.02 ± 8.07	16	5.21 ± 0.79	26

Note: Data are presented as mean ± sd. Sensilla abbreviation: SB, sensilla basiconica; SC, sensilla chaetica; SD, sensilla digitiformia; SE, sensilla epipharyngea; SP, sensilla placodea; ST, sensilla styloconica.

## Data Availability

The original contributions presented in this study are included in the article/Supplementary Material. Further inquiries can be directed to the corresponding authors.

## Supplementary Materials

The following supporting information can be downloaded at: https://www.mdpi.com/article/10.3390/insects17030345/s1. Table S1. Raw measurement data of sensilla on the antennae and mouthparts of *Conogethes punctiferalis* larvae.
